# Elevated Serum Lactate Dehydrogenase Predicts Unfavorable Outcomes After rt-PA Thrombolysis in Ischemic Stroke Patients

**DOI:** 10.3389/fneur.2022.816216

**Published:** 2022-04-06

**Authors:** Huijuan Jin, Rentang Bi, Jichuan Hu, Da Xu, Ying Su, Ming Huang, Qiwei Peng, Zhifang Li, Shengcai Chen, Bo Hu

**Affiliations:** ^1^Department of Neurology, Union Hospital, Tongji Medical College, Huazhong University of Science and Technology, Wuhan, China; ^2^Department of Neurology, People's Hospital of Dongxihu District, Wuhan, China; ^3^Department of Neurology, Hubei Provincial Hospital of Integrated Chinese and Western Medicine, Hubei University of Chinese Medicine, Wuhan, China

**Keywords:** ischemic stroke, lactic dehydrogenase, rt-PA, prognosis of prognosis, something, bio-marker

## Abstract

**Background and Purpose:**

Currently, acute ischemic stroke (AIS) is one of the most common and serious diseases in the world and is associated with very high mortality and morbidity even after thrombolysis therapy. This study aims to research the relationship between lactic dehydrogenase (LDH) and prognosis in AIS patients treated with intravenous rtPA.

**Method:**

This study (a Multicenter Clinical Trial of Revascularization Treatment for Acute Ischemic Stroke, TRAIS) included 527 AIS patients in 5 cooperative medical institutions in China from January 2018 to February 2021. The primary outcome was major disability and death within 3 months (mRS score of 3–6), and the secondary outcomes were early neurological improvement (ENI), early neurological deterioration (END), moderate-severe cerebral edema (CE), and symptomatic intracranial hemorrhage (sICH).

**Results:**

The mean age of the 527 patients was 65.6 ± 11.7 years, and the median baseline NIHSS score was 4 (interquartile range, 2–7). The median serum LDH level was 184 U/L (interquartile range, 163–212 U/L). In total, 287 (54.5%) patients acquired ENI, 68 (13.0%) patients suffered END, 53 (12.1%) patients were observed with moderate-severe CE, and 28 (6.2%) patients showed sICH. Within 3 months, 127 (25.15%) patients experienced the primary outcome and 42 (8.3%) patients died. Serum LDH levels before thrombolysis showed an independent association with the risk of primary outcome [adjusted odds ratio, 3.787; (95% CI, 1.525–9.404); *P* = 0.014]. When log-transformed LDH increased each standard deviation, the risk of primary outcome was raised by 80.1% (95% CI, 28.9–251.7%). A positive linear dependence between the risk of primary outcome and serum LDH levels (*P* of linearity = 0.0248, *P* of non-linearity = 0.8284) was shown in multivariable-adjusted spline regression models. Pre-thrombolysis LDH quartile also provided a conventional risk model and significant improvement of the prediction for clinical outcomes, with a net reclassification improvement index (NRI) = 41.86% (*P* < 0.001) and integrated discrimination improvement (IDI) = 4.68% (*P* < 0.001).

**Conclusions:**

Elevated serum LDH levels predicted unfavorable clinical outcomes after intravenous thrombolysis in AIS patients.

## Introduction

Acute ischemic stroke (AIS) is currently one of the most disabling and lethal diseases in the world (1). Intravenous thrombolysis with recombinant tissue plasminogen activator (rt-PA) as well as endovascular therapy is the most effective primary modality of therapy (2, 3). However, a significant number of patients who receive intravenous rt-PA therapy still face the threat of complications such as hemorrhage transformation and cerebral edema (CE), with unfavorable recovery of neurological function (3, 4). Therefore, it is significant to explore novel prognostic biomarkers in AIS patients for clinical decision making.

Lactate dehydrogenase (LDH) is a critical enzyme of the anaerobic metabolic pathway and is mainly distributed in the cytoplasm and mitochondria of various tissues, including the brain, heart, liver, and lung under physiological conditions (5, 6). Once the tissue is injured, LDH is released to the extracellular space and leads to an increased serum LDH level. Thus, LDH has been regarded as a biomarker of both tissue injury and prognosis in many diseases, including acute myocardial infarction, acute hepatitis, and acute lung injury (7–10). Presumably, LDH gets rapidly upregulated in brain parenchyma in response to ischemia and hypoxia after AIS, and leaks into circulating blood with the aggravation of cerebral infarction and peripheral edema. LDH has been observed to be released from brain tissue in animal models of brain injury, including ischemic stroke, and has been applied as a marker of brain tissue injury in basial experiments (11–13). Clinically, LDH is found to be elevated in the serum and cerebrospinal fluid of patients with ischemic stroke and related to the occurrence of stroke (14).

The relationship between LDH levels and clinical outcomes in AIS patients has never been thoroughly studied (15, 16). This study aimed to analyze the correlation between serum LDH and clinical outcomes in patients receiving intravenous rt-PA treatment.

## Materials and Methods

### Study Population

We conducted a retrospective study (Multicenter Clinical Trial of Revascularization Treatment for Acute Ischemic Stroke, TRAIS) using all consecutive AIS cases for thrombolysis in 5 transregional cooperative medical institutions in China, including Wuhan Union Hospital, the West Branch of Wuhan Union Hospital, Hefeng People's Hospital, the People's Hospital of Dongxihu District, and Yichang Central Hospital. Patients admitted from 1 January 2018 to 1 February 2021 were used in the analysis.

The ethics of this study are in line with the principles expressed in the Declaration of Helsinki. The local institutional review board approved all aspects of the study (ChiCTR2000033456).

### Inclusion and Exclusion Criteria

Patients were selected according to the following criteria: (1) clinically confirmed AIS; (2) rt-PA injections consistent with the indications for thrombolytic therapy; and (3) patients aged 18 years or older. Exclusion criteria included: (1) AMI, severe infectious diseases, terminal cancer, hematological disease, hepatic or renal disease, recent major trauma or surgery; (2) mental disorders, severe cognitive dysfunction; and (3) incomplete clinical data. All available inpatient data, including history, clinical tests, laboratory tests, diagnostic tests, imaging studies, and discharge diagnoses, were used for the diagnosis of the above diseases.

### Treatment Administration

All the patients received thrombolytic treatment in line with written institutional guidelines. The time window for thrombolysis was extended and limited up to 9 h guided by perfusion imaging (17, 18). Intravenous rt-PA (administered at a standard dose of 0.9 mg/kg body weight) was given according to the procedure recommended by the European Stroke organization guidelines in 2018 (19). In total, 10% of the total dose was given as the first dose and the remaining dose was given within the next hour. Continuous monitoring and evaluation were conducted during thrombolysis. After thrombolysis was completed, the patient was transferred to the Neurological ICU for intensive nursing.

### Laboratory Determinations

Serum LDH levels were adopted from each patient both in the emergency ward before thrombolysis and within 1–3 days in the inpatient ward. We ensured that the serum sample did not develop hemolysis prior to testing.

### Clinical Assessment

We retrieved: (1) clinical assessment from the National Institute of Health stroke scale (NIHSS) score evaluated both on admission and 24 h after thrombolysis, baseline blood pressure, time from onset to treatment (OTT); (2) demographic information, including gender and age; (3) vascular risk factors such as alcohol drinking, cigarette smoking, hypertension, diabetes mellitus, coronary heart disease, and previous stroke; (5) auxiliary examination, including multimodal computerized tomography (CT), magnetic resonance imaging (MRI), cervical vascular ultrasound, and echocardiography of patients. All patients underwent the same level of standardized assessment prior to discharge and were given a personalized rehabilitation plan.

### Clinical Outcomes

Participants were followed-up by modified Rankin Scale (mRS) scores at 3 months after intravenous thrombolysis by trained neurologists, who were unaware of treatment assignment. This study was conducted in January 2018, and follow-up work was completed in May 2021.

Clinical outcomes were determined as: (1) 3-month death or major disability (mRS, 3–6); (2) 3-month mortality (mRS score of 6); (3) cerebral edema, according to the Safe Implementation of Thrombolysis in Stroke-Monitoring Study (SITS-MOST) criteria (20), we classified those with a swelling area >1/3 of hemicerebrum or midline deviation as moderate-severe cerebral edema, based on CT or MRI within 1–3 days after thrombolysis; (4) symptomatic intracranial hemorrhage (sICH), using the National Institute of Neurological Disorders and Stroke (NINDS) criteria (21); (5) early neurological improvement (ENI), defined as NIHSS score decrease of ≥ 4 or complete recovery within 24 h after thrombolysis; (6) early neurological deterioration (END), defined as NIHSS score increase of ≥ 4 within 24 h after thrombolysis.

### Statistical Analysis

In all processed data, variables that fit the normal distribution are recorded as the mean and standard deviation, while variables that are not normally distributed are recorded as the median, quartile, and distribution range. For dichotomous variables, we give the quantity and distribution ratio. According to the normality of the data distribution, independent samples 2-tailed *T*-test, Mann-Whitney *U*-test, or χ^2^ test are used for the dichotomous variables between groups.

The level of LDH was converted to a categorical variable according to the quartile to facilitate the comparison of differences between the two extreme groups. At the same time, LDH showed acceptable normality after natural logarithmic conversion. Concomitant variables in the multinomial logistic regression analysis include sex, age, OTT time, current cigarette smoking, alcohol drinking, history of stroke, hypertension, diabetes mellitus, dyslipidemia, and coronary heart disease and baseline NIHSS score. The statistical significance was set to the probability value < 0.05. All the above analyses are performed with SPSS 25.0 for Windows.

R (version 4.0.3) software, Net reclassification improvement (NRI), and comprehensive discriminant improvement (IDI) were used to evaluate the net benefit of reclassification for LDH survival and prediction of malignant edema. In addition, with MedCalc (version 20.0.3) software, we used receiver operating characteristic (ROC) curves to compare the overall discriminative ability between pre-thrombolysis and post-thrombolysis LDH for outcomes.

### Data Availability

The data used for the analysis of the study results are available from the corresponding author on reasonable request.

## Results

### Baseline Characteristics

As shown in Supplementary Figure 1, there was a total of 718 AIS patients treated with intravenous rt-PA in our medical centers, 55 patients were excluded for AMI, cancer, severe infection, or serious systemic disease, and 136 patients had a lack of clinical data, blood samples lost, or loss of follow-up. Finally, the data of 527 patients were applied to the subsequent analysis for this study. The mean age was 65.6 years (SD ± 11.7 years, range, 33–95 years), and 66.8% of patients were male. The median baseline NIHSS score was 4 (interquartile range, 2–7). The median serum LDH before thrombolysis was 190 U/L (interquartile range,163–212 U/L). All baseline characteristics among LDH quartiles are provided in Table 1. It was shown that higher serum LDH quartiles were associated with female patients, higher baseline NIHSS scores, and larger final infarct volume, while lower LDH levels are related to small-artery occlusion.

**Table 1:** Baseline characteristics of the study participants among LDH quartiles.

**Characteristics**	**Total**	**LDH before thrombolysis, U/L**				***P* Value for trend**
		**Q1 (<163)**	**Q2 (163–184)**	**Q3 (185–212)**	**Q4 (>212)**	
Patients, *n*	527	131	132	132	132	
**Demographic**						
Age, y	65.6 ± 11.7	65.28 ± 11.4	64.7 ± 12.2	65.5 ± 10.8	67.2 ± 12.3	0.654
Sex, male	348 (66.8%)	107 (79.3%)	99 (74.4%)	70 (55.1%)	72 (56.2%)	<0.001
**Vascular risk factors, %**						
Hypertension	363 (68.9%)	98 (72.1%)	87 (65.4%)	85 (66.9%)	93 (71.0%)	0.345
Diabetes mellitus	119 (22.6%)	38 (27.9%)	27 (20.3%)	29 (22.8%)	25 (19.1%)	0.1
Dyslipidemia	146 (27.7%)	34 (25.0%)	40 (30.1%)	41 (32.3%)	31 (23.7%)	0.406
Previous ischemic stroke	74 (14.0%)	19 (14.0%)	19 (14.3%)	13 (10.2%)	23 (17.6%)	0.978
Previous intracerebral hemorrhage	15 (2.8%)	5 (3.7%)	3 (2.3%)	3 (2.3%)	4 (3.1%)	0.536
Coronary heart disease	64 (12.1%)	15 (11.0%)	11 (8.3%)	19 (15.0%)	19 (14.5%)	0.636
Atrial Fibrillation	68 (12.9%)	6 (4.4%)	11 (8.3%)	19 (14.6%)	32 (24.8%)	0.001
Current cigarette smoking	150 (28.4%)	47 (34.6%)	48 (36.1%)	29 (22.8%)	26 (19.8%)	0.079
Current alcohol drinking	105 (19.8%)	21 (15.4%)	37 (27.8%)	22 (17.3%)	25 (19.1%)	0.129
**Clinical assessment**						
Baseline NIHSS score	4 (2–7)	3 (1–6)	3 (2–6)	3 (1–7)	6 (2–12)	0.014
Baseline systolic BP, mm Hg	148 (135–160)	147 (134–160)	150 (136–158)	146 (135–160)	150 (138–164)	0.359
Baseline diastolic BP, mm Hg	84 (76–92)	84 (78–90)	85 (77–92)	83 (74–92)	87 (77–95)	0.411
Admission blood glucose, mmol/L	6.7 (5.6–8.2)	6.69 (5.8–8.23)	7.1 (5.78–8.2)	6.5 (5.3–7.6)	6.7 (5.45–9.25)	0.662
OTT, minute	198 (140–255)	203 (150–256)	189 (130–255)	205 (152–259)	185 (140–237)	0.821
Infarct size, ml	0.65 (0–8.63)	0.12 (0–3.6)	0.19 (0–3.2)	0.28 (0–9.81)	8.39 (0–52.31)	0.009
Small size (<1 ml)	236 (54%)	78 (64.5%)	64 (59.3%)	61 (56%)	33 (33.1%)	0.007
Middle size (1–20 ml)	129 (29.5%)	31 (25.6%)	35 (32.4%)	32 (29.4%)	31 (31.3%)	0.269
Large size (>20 ml)	72 (16.5%)	12 (9.9%)	9 (8.3%)	16 (14.7%)	35 (35.4%)	0.022
**Stroke subtype, %**						
Large–artery atherosclerosis	94 (17.8%)	20 (14.7%)	21 (15.8%)	21 (16.5%)	32 (24.4%)	0.247
Cardio–embolism	53 (10.1%)	10 (7.4%)	4 (3.0%)	13 (10.2%)	26 (19.8%)	0.186
Small–artery occlusion	219 (41.6%)	69 (50.7%)	59 (44.4%)	61 (48.0%)	30 (22.9%)	0.013
Stroke of undetermined cause	161 (30.6%)	37 (27.2%)	49 (36.8%)	32 (25.2%)	43 (32.8%)	0.317
**Lesion location**, ***n*** **(%)**						
Anterior circulation	434 (86.1%)	111 (83.5%)	110 (88.7%)	109 (87.2%)	104 (85.2%)	0.328
Posterior circulation	70 (13.9%)	22 (16.5%)	14 (11.3%)	16 (12.8%)	18 (14.8%)	0.328

*BP indicates blood pressure; NIHSS, National Institutes of Health Stroke Scale*.

### Serum LDH Levels at Baseline and Clinical Outcomes of AIS Patients Treated With Intravenous rt-PA

Within 3 months, 127 patients (25.1%) experienced primary outcomes (85 with severe disability and 42 with death; Table 2), and the cumulative rates for the four serum LDH quartiles (Q1 to Q4) were 15.7, 19.2, 18.3, and 48.4% (*P* < 0.001). After intravenous thrombolysis, 287 (54.5%) patients acquired ENI, 68 (13.0%) patients suffered END, 53 (12.1%) patients were found with moderate-severe CE, and 28 (6.2%) patients showed sICH.

**Table 2:** Odds ratio and 95% CI of clinical outcomes for serum LDH before thrombolysis.

	**LDH before thrombolysis, U/L (*****n*** **=** **527)**				***P* value for trend**	**Each SD increase of Log-LDH**
	**Q1 (<163)**	**Q2 (163–184)**	**Q3 (185–212)**	**Q4 (>212)**		
**Primary outcome: death or major disability (mRS score of 3–6)**						
No. of cases, *n* (%)	21 (16.0%)	25 (18.9%)	22 (16.7%)	59 (44.7%)	127 (24.1%)	
Model 1	Ref	1.123 (0.568, 2.217)	1.229 (0.614, 2.46)	4.704 (2.45, 9.033)	<0.001	2.145 (1.651, 2.786)
Model 2	Ref	1.497 (0.671, 3.338)	1.202 (0.506, 2.854)	3.753 (1.645, 8.565)	0.006	1.816 (1.329, 2.481)
**Secondary outcomes**						
ENI						
No. of cases, *n* (%)	94 (71.7%)	81 (61.4%)	69 (52.3%)	43 (32.6%)	287 (54.5%)	
Model 1	Ref	0.946 (0.501, 1.787)	0.590 (0.323, 1.078)	0.218 (0.119, 0.399)	<0.001	0.496 (0.389, 0.633)
Model 2	Ref	0.888 (0.466, 1.691)	0.576 (0.312, 1.062)	0.261 (0.14, 0.489)	<0.001	0.540 (0.419, 0.697)
END						
No. of cases, *n* (%)	3 (2.5%)	7 (5.3%)	7 (5.3%)	54 (41.9%)	68 (13.0%)	
Model 1	Ref	1.754 (0.147, 20.927)	2.308 (0.19, 27.974)	16.605 (1.964, 140.368)	0.001	2.970 (1.896, 4.653)
Model 2	Ref	2.011 (0.163, 24.785)	1.944 (0.153, 24.641)	8.699 (0.975, 77.574)	0.062	2.258 (1.362, 3.743)
moderate-severe CE						
No. of cases, *n* (%)	5 (3.8%)	7 (5.3%)	13 (9.8%)	28 (21.2%)	53 (10.1%)	
Model 1	Ref	1.499 (0.435, 5.161)	3.673 (1.184, 11.395)	7.332 (2.493, 21.560)	<0.001	2.440 (1.690, 3.524)
Model 2	Ref	2.158 (0.561, 8.291)	3.834 (1.079, 13.619)	5.567 (1.650, 18.786)	0.038	2.106 (1.408, 3.150)
sICH						
No. of cases, *n* (%)	7 (5.3%)	2 (1.5%)	9 (6.8%)	10 (7.5%)	28 (5.3%)	
Model 1	Ref	0.228 (0.043, 1.204)	0.804 (0.382, 3.456)	0.861 (0.276, 2.690)	0.266	0.999 (0.828, 1.205)
Model 2	Ref	0.278 (0.053, 1.464)	1.008 (0.318, 3.197)	0.482 (0.141, 1.649)	0.267	0.750 (0.473, 1.189)

*Model 1, adjusting for age, sex, admission blood glucose, time from onset to treatment, baseline systolic blood pressure, current smoking, alcohol consumption, history of stroke, hypertension, diabetes mellitus, dyslipidemia, coronary heart disease, and atrial fibrillation. Model 2, further adjusting for baseline National Institutes of Health Stroke Scale score based on adjusting for factors in model 1. ENI, early neurological improvement; CE, cerebral edema; sICH, symptomatic intracranial hemorrhage*.

**Figure 1:**
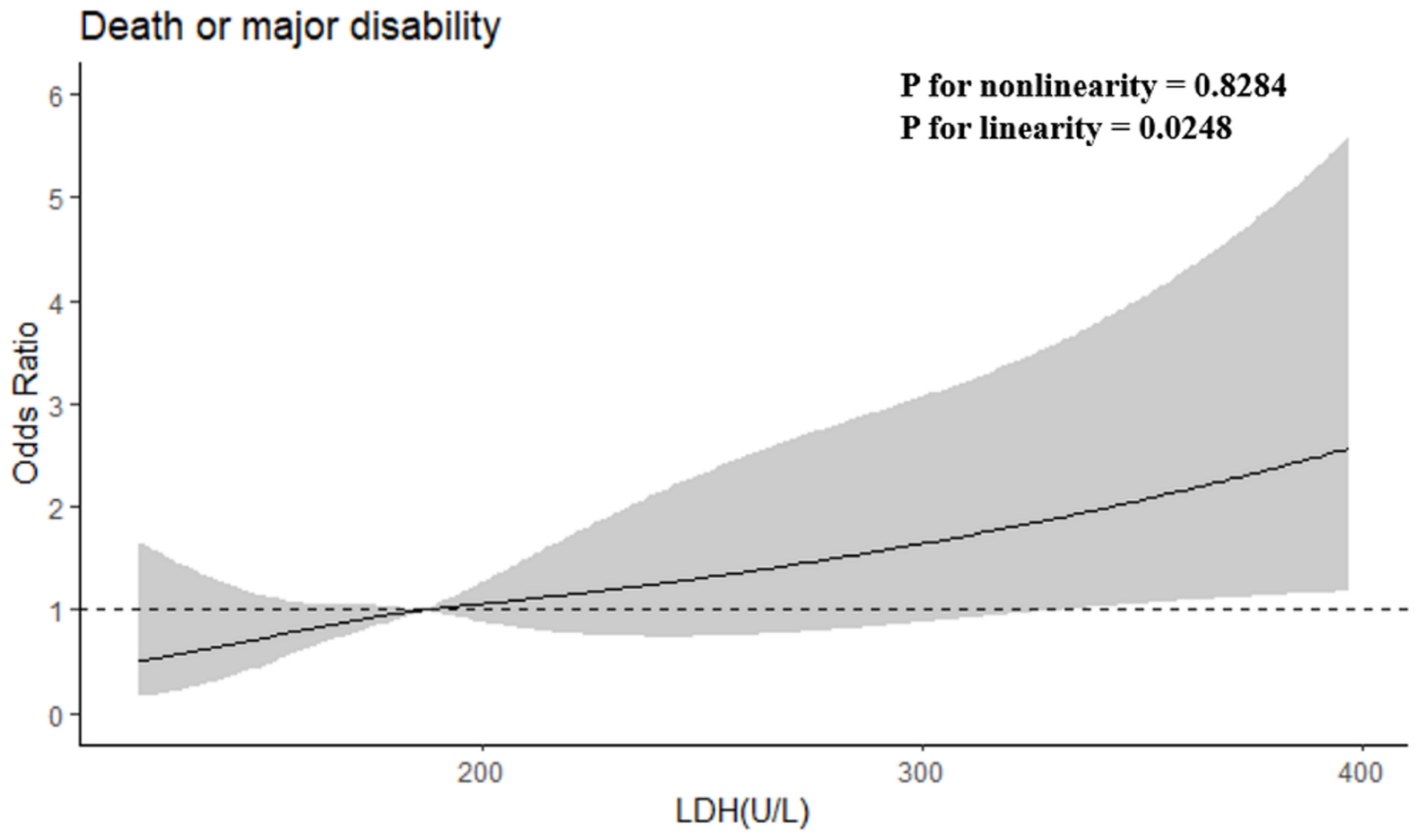
Adjusted odds ratios of primary outcome according to serum LDH. OR and 95% CI derived from restricted cubic spline regression, with knots placed at the 5th, 35th, 65th, and 95th percentiles of LDH. OR adjusted for the same variables as model 2 in Table 2.

After adjusting baseline NIHSS score and other potential confounders in model 2, when the highest and lowest quartiles (Q4 and Q1) were compared, serum LDH levels before thrombolysis showed independently associated with the risk of primary outcome (odds ratio, 3.753; 95% CI, 1.645–8.565; *P* for trend = 0.002). The patients in the highest quartile of LDH showed a 2.787-fold increased risk of death or disability compared with those in the lowest quartile. Moreover, when log-transformed LDH increased each standard deviation (SD), the risk of primary outcome got raised by 81.6% (95% CI, 32.9–248.1%) in model 2. Furthermore, a positive linear dose-response relationship between the risk of the primary outcome and pre-thrombolysis serum LDH was shown in multivariable-adjusted spline regression models (*P* of linearity = 0.0248, *P* of non-linearity = 0.8284; Figure 1).

### Incremental Predictive Value of Serum LDH for Clinical Outcomes of AIS Patients Treated With Intravenous rt-PA

We further examined the incremental predictive value of serum LDH before thrombolysis and the conventional model that includes all risk factors in Model 2 for the clinical outcomes of AIS patients treated with intravenous rt-PA. As shown in Table 3, adding pre-thrombolysis LDH quartile significantly improved the prediction for the risk of primary outcome of the conventional risk model, with NRI = 41.86% (*P* < 0.001) and IDI = 4.68% (*P* < 0.001). When log-transformed LDH was added to the model, the NRI and IDI for primary outcome were 41.14% (95%CI, 21.33–60.95%) and IDI of 3.08% (95%CI, 0.91–5.26%), respectively.

**Table 3:** Reclassification and discrimination Statistics for clinical outcomes by serum LDH before thrombolysis.

**Clinical outcomes**		**Continuous NRI, %**		**IDI, %**	
	**Model**	**Estimate (95% CI)**	***P* value**	**Estimate (95% CI)**	***P* value**
**Primary outcome**					
Death or major disability (mRS score of 3–6)	Conventional model	Reference		Reference	
	Conventional model + LDH quartile	0.4286 (0.2263, 0.611)	<0.001	0.0468 (0.0276, 0.066)	<0.001
	Conventional model	Reference		Reference	
	Conventional model + log-transformed LDH	0.4114 (0.2133, 0.6095)	<0.001	0.0308 (0.0091, 0.0526)	0.005
**Secondary outcomes**					
END	Conventional model	Reference		Reference	
	Conventional model + LDH quartile	0.754 (0.3754, 1.1326)	<0.001	0.0337 (0.0135, 0.0538)	<0.001
	Conventional model	Reference		Reference	
	Conventional model + log-transformed LDH	0.6445 (0.2017, 1.0872)	0.004	0.0564 (0.004, 0.1129)	0.04
ENI	Conventional model	Reference		Reference	
	Conventional model + LDH quartile	0.6942 (0.4436, 0.9447)	<0.001	0.0877 (0.0576, 0.1177)	<0.001
	Conventional model	Reference		Reference	
	Conventional model + log-transformed LDH	0.4138 (0.2232, 0.6043)	<0.001	0.0538 (0.0306, 0.6043)	<0.001
Moderate-severe CE	Conventional model	Reference		Reference	
	Conventional model + LDH quartile	0.4512 (0.2607, 0.6418)	<0.001	0.0852 (0.0586, 0.1119)	<0.001
	Conventional model	Reference		Reference	
	Conventional model + log-transformed LDH	0.6296 (0.3498, 0.9093)	<0.001	0.0353 (0.0086, 0.0619)	0.009

*Conventional model was model 2 in Table 2, including age, sex, admission blood glucose, time from onset to hospitalization, baseline systolic blood pressure, current smoking, alcohol consumption, history of stroke, hypertension, diabetes mellitus, dyslipidemia, coronary heart disease, atrial fibrillation, and National Institutes of Health Stroke Scale score at baseline. IDI, indicates integrated discrimination index; and NRI, net reclassification improvement*.

### Subgroup Analysis of the Association Between Serum LDH Before Thrombolysis and Primary Outcome

On the primary outcome, we conducted subgroup analyses to examine the potential modified effect of prespecified factors. Stratified by baseline systolic BP, age, gender, baseline NIHSS score, cigarette smoking, alcohol drinking, history of hypertension, and dyslipidemia, no statistically significant interaction between pre-thrombolysis serum LDH and these interesting factors were observed (all *P* values of interaction are >0.05; Table 4).

**Table 4:** Subgroup analysis of the association between LDH before thrombolysis and primary outcome (death or major disability).

**Subgroup**	**Primary outcome: death or major disability (mRS score of 3–6)**	
	**OR (95%CI)**	***P* of interaction**
Age, y		
<60	2.424 (1.212, 4.849)	0.542
60–69	1.81 (1.294, 2.531)	
≥70	1.595 (0.876, 2.902)	
Sex		
men	2.064 (1.381, 3.084)	0.099
women	1.778 (0.695, 4.552)	
OTT, h		
≤ 3	2.341 (1.33, 4.121)	0.434
3–4.5	1.65 (0.936, 2.907)	
> 4.5	1.518 (0.515, 4.477)	
SBP, mmHg		
<160	1.767 (1.204, 2.594)	0.875
≥160	3.859 (0.969, 15.358)	
Admission NIHSS score		
<4	1.958 (1.117, 3.434)	0.103
≥4	1.84 (1.269, 2.667)	
Admission blood glucose		
<7.0	1.677 (0.931, 3.02)	0.236
<7.0	2.118 (1.393, 3.221)	
Hypertension		
NO	1.940 (1.001, 3.759)	0.779
YES	1.777 (1.134, 2.784)	
Diabetes mellitus		
NO	2.03 (1.36, 3.029)	0.2
YES	1.791 (0.778, 4.124)	
Dyslipidemia		
NO	1.391 (0.885, 2.187)	0.057
YES	3.353 (1.433, 7.843)	
Previous ischemic stroke		
NO	1.779 (1.261, 2.51)	0.788
YES	8.038 (0.181, 356.805)	
Previous intracerebral hemorrhage	
NO	1.897 (1.336, 2.643)	0.36
YES	1.197 (0.003, 11.364)	
Coronary heart disease		
NO	1.843 (1.288, 2.637)	0.845
YES	2.93 (0.43, 18.144)	
Atrial fibrillation		
NO	1.996 (1.275, 1.481)	0.171
YES	1.983 (0.736, 5.347)	
Current cigarette smoking		
NO	1.714 (1.131, 2.597)	0.419
YES	2.708 (1.287, 5.696)	
Current alcohol drinking		
NO	1.75 (1.206, 2.539)	0.859
YES	2.045 (0.858, 4.873)	
Stroke subtype		
Atherothrombotic	1.973 (0.735, 5.295)	0.651
Cardioembolic	2.014 (0.713, 6.221)	
Lacunar	1.987 (1.008, 3.917)	
Lesion location		
Anterior circulation	1.917 (1.229, 2.991)	0.118
Posterior circulation	1.045 (0.492, 2.22)	

*Interactions between serum LDH and interesting factors on the primary outcome were tested by the likelihood ratio test with adjustment for the same variables as model 2 in Table 2 unless the variable was used as a subgroup variable. NIHSS indicates National Institutes of Health Stroke Scale; and OR, odds ratio*.

### Serum LDH Levels Within 1–3 Days After Thrombolysis and 3-Month Prognosis

The median LDH levels within 1–3 days after thrombolysis were 196 (quartiles, 173–230) U/L, significantly exceeding LDH levels before thrombolysis (*P* < 0.001). Therefore, we further analyzed the predictive power of LDH levels within 1–3 days on primary outcomes and 3-month mortality. In model 2, the OR of primary outcome for the Q4 vs. Q1 is 19.876 (95%CI, 4.626–85.397). Each SD increase in log-transformed LDH was associated with a 320.8% (121, 701.4%) increased risk of primary outcome (Supplementary Table 1). Furthermore, ROC curves were used to compare the overall discriminative ability between LDH before thrombolysis and within 1–3 days after thrombolysis for the 3-month outcome and to calculate optimal cut-off values which represent the highest sum of the specificity and sensitivity. Post-thrombolysis LDH had an obvious advantage in AUC relative to pre-thrombolysis LDH for predicting primary outcomes (Table 5).

**Table 5:** AUC for serum LDH levels before and within 1–3 days after thrombolysis of 3-month prognosis.

	**AUC**	**95% CI**	**Optimal cutoff value**	**Specificity**	**Sensitivity**	**Youden index**
ROC curves for major disability and death
LDH before thrombolysis	0.692	(0.602, 0.781)	205	75.37	51.39	0.2676
LDH within 1–3 days after thrombolysis	0.762	(0.682, 0.842)	235	86.49	53.13	0.3961

*AUC, Area under the ROC curve*.

## Discussion

Our study shows that (1) elevated serum LDH levels on admission were significantly associated with ENI, END, cerebral edema, and 3-month outcomes in AIS patients. (2) Serum LDH levels before thrombolysis have additional predictive incremental value for traditional models that include baseline NIHSS scores. (3) Serum LDH levels within 1–3 days after thrombolysis are more predictive of 3-month outcomes than baseline levels.

LDH, as an enzyme necessary for anaerobic metabolism, is localized and restricted intracellular unless local tissue is injured. The brain should be one of the sources of serum LDH after AIS. On one hand, intracellular LDH is upregulated for energy utilization and adaption to the ischemic and hypoxic environment results from occlusion of cerebral arteries (5), which may occur in all injured brain cells, including neurons, astrocytes, microglia, and so on. Elevated LDH was detected in the brain cells during hypoxia and reoxygenation (22). On the other hand, damage or death of brain cells allows LDH to be released into the extracellular space and then into the peripheral circulation through the damaged BBB. Furthermore, it has also been found that extracellular lactic acid, the catalytic products of LDH, stimulates vascular endothelial cells to express inflammatory factor IL-8 and vascular endothelial growth factor in tumor study (23–26), which possibly promote local inflammation and angiogenesis, contributing to the BBB destruction and cerebral edema in ischemic stroke. Thus, we hypothesized that the elevated serum LDH responsibly reflects the severity of brain tissue injury. In this study, we validated that serum LDH levels are associated with cerebral infarct size and cerebral edema and predicted neurological changes and 90-day outcomes.

During 1–3 days after thrombolysis, the cerebral infarct size may continue to expand with an acute progression of cerebral edema, leading to a continuous increasing LDH level. On the other hand, as the fact that BBB remains relatively intact within 24 h, the leakage of LDH to peripheral blood may be limited to a certain extent. Therefore, serum LDH levels within 1–3 days after thrombolysis possibly more accurately reflected cerebral damage after intravenous thrombolysis, thereby becoming a better predictor of 90-day outcomes, while serum LDH levels before thrombolysis may provide additional assistance in decision making for thrombolytic therapy.

Recently, elevated LDH levels were reported to be associated with 30-day mortality in AIS patients with COVID-19 (27). However, the elevated serum LDH levels likely resulted from pneumonia, which has been reported in other published articles (28). In contrast, we deliberately exclude other diseases that potentially increase serum LDH levels.

The limitation of this study is that (1) all the patients in this study were Chinese, so there is a lack of validation in non-Chinese patients; (2) the sample size is relatively small; (3) we did not record serum LDH levels on a continuous basis after intravenous thrombolysis.

In conclusion, serum LDH could be an extremely promising prognostic marker in AIS patients treated with intravenous rt-PA.

## Data Availability Statement

The raw data supporting the conclusions of this article will be made available by the authors, without undue reservation.

## Ethics Statement

The studies involving human participants were reviewed and approved by Ethics Committee of Huazhong University of Science and Technology. Written informed consent for participation was not required for this study in accordance with the national legislation and the institutional requirements.

## Author Contributions

RB, SC, and JH conducted the data analysis and wrote the manuscript. JH, DX, YS, and MH helped with the data collection and literature search. BH together with HJ designed this study and directed the writing of the manuscript. All authors contributed to the article and approved the submitted version.

## Funding

This work was supported by the National Key Research and Development Program of China (2018YFC1312200 to BH), the National Natural Science Foundation of China (Grants: 82090044 and 81820108010 to BH, and 81671147 to HJ), and Major Refractory Diseases Pilot Project of Clinical Collaboration with Chinese and Western Medicine (SATCM-20180339 to BH).

## Conflict of Interest

The authors declare that the research was conducted in the absence of any commercial or financial relationships that could be construed as a potential conflict of interest.

## Publisher's Note

All claims expressed in this article are solely those of the authors and do not necessarily represent those of their affiliated organizations, or those of the publisher, the editors and the reviewers. Any product that may be evaluated in this article, or claim that may be made by its manufacturer, is not guaranteed or endorsed by the publisher.

## Abbreviations

AIS: acute ischemic stroke
rt-PA: recombinant tissue plasminogen activator
CE: cerebral edema
LDH: lactate dehydrogenase
AMI: acute myocardial infarction
NIHSS: National Institute of Health stroke scale
OTT: time from onset to treatment (OTT)
CT: computerized tomography
MRI: magnetic resonance imaging
mRS: modified Rankin Scale
SITS-MOST: the Safe Implementation of Thrombolysis in Stroke-Monitoring Study
NINDS: National Institute of Neurological Disorders and Stroke
ENI: early neurological improvement
END: early neurological deterioration
OR: odds ratio
IDI: integrated discrimination improvement
NRI: net reclassification improvement
ROC: receiver operating characteristic.
