# Supplementary material for: Elevated Serum Lactate Dehydrogenase Predicts Unfavorable Outcomes After rt-PA Thrombolysis in Ischemic Stroke Patients
Source: Front Neurol. 2022 Apr 6;13:816216. doi: 10.3389/fneur.2022.816216 (PMC9019114; doi:10.3389/fneur.2022.816216)
Supplement: Supplementary file 1 [file Table_1.DOCX]

Supplemental Table1. Odds ratio and 95% CI of clinical outcomes for quartile of LDH within 3 days after thrombolysis.

|  | LDH after thrombolysis, U/L (n=283) | | | | *P* Value for Trend | Each SD Increase of Log-LDH |
| --- | --- | --- | --- | --- | --- | --- |
|  | Q1 (<173) | Q2 (173–196) | Q3 (197–230) | Q4(＞230) |  |  |
| Primary outcome: death or major disability (mRS score of 3–6) | | | |  |  |  |
| No. of cases, n (%) | 8 (11.1%) | 13 (17.6%) | 15 (21.4%) | 47 (66.2%) | 83 (28.8%) |  |
| Model 1 | ref | 3.38 (0.921, 12.405) | 3.928 (1.122, 13.745) | 39.105 (10.9, 140.288) | ＜0.001 | 5.77 (3.311, 10.058) |
| Model 2 | ref | 2.511 (0.569, 11.082) | 2.171 (0.507, 9.289) | 19.876 (4.626, 85.397) | ＜0.001 | 4.208 (2.21, 8.014) |
| Secondary outcome: Death (mRS score of 6) | | |  |  |  |  |
| No. of cases, n (%) | 0 (0%) | 1 (1.4%) | 4 (5.7%) | 22 (31.0%) | 27 (9.4%) |  |
| Model 1 | ref | 1.234 (0.158, 9.648) | 3.161 (0.545, 18.335) | 21.457 (4.286, 107.42) | ＜0.001 | 5.591 (2.878, 10.86) |
| Model 2 | ref | 1.127 (0.136, 9.323) | 2.626 (0.415, 16.624) | 11.639 (2.221, 61) | 0.002 | 4.069 (2.177, 7.607) |

Model 1, adjusting for age, sex, time from onset to treatment, baseline systolic blood pressure, current smoking, alcohol consumption, history of stroke, hypertension, diabetes mellitus, dyslipidemia, and coronary heart disease. Model 2, further adjusting for baseline National Institutes of Health Stroke Scale score based on adjusting for factors in model 1.
