# Supplementary figures and images for: Elevated Serum Lactate Dehydrogenase Predicts Unfavorable Outcomes After rt-PA Thrombolysis in Ischemic Stroke Patients
Source: Front Neurol. 2022 Apr 6;13:816216. doi: 10.3389/fneur.2022.816216 (PMC9019114; doi:10.3389/fneur.2022.816216)

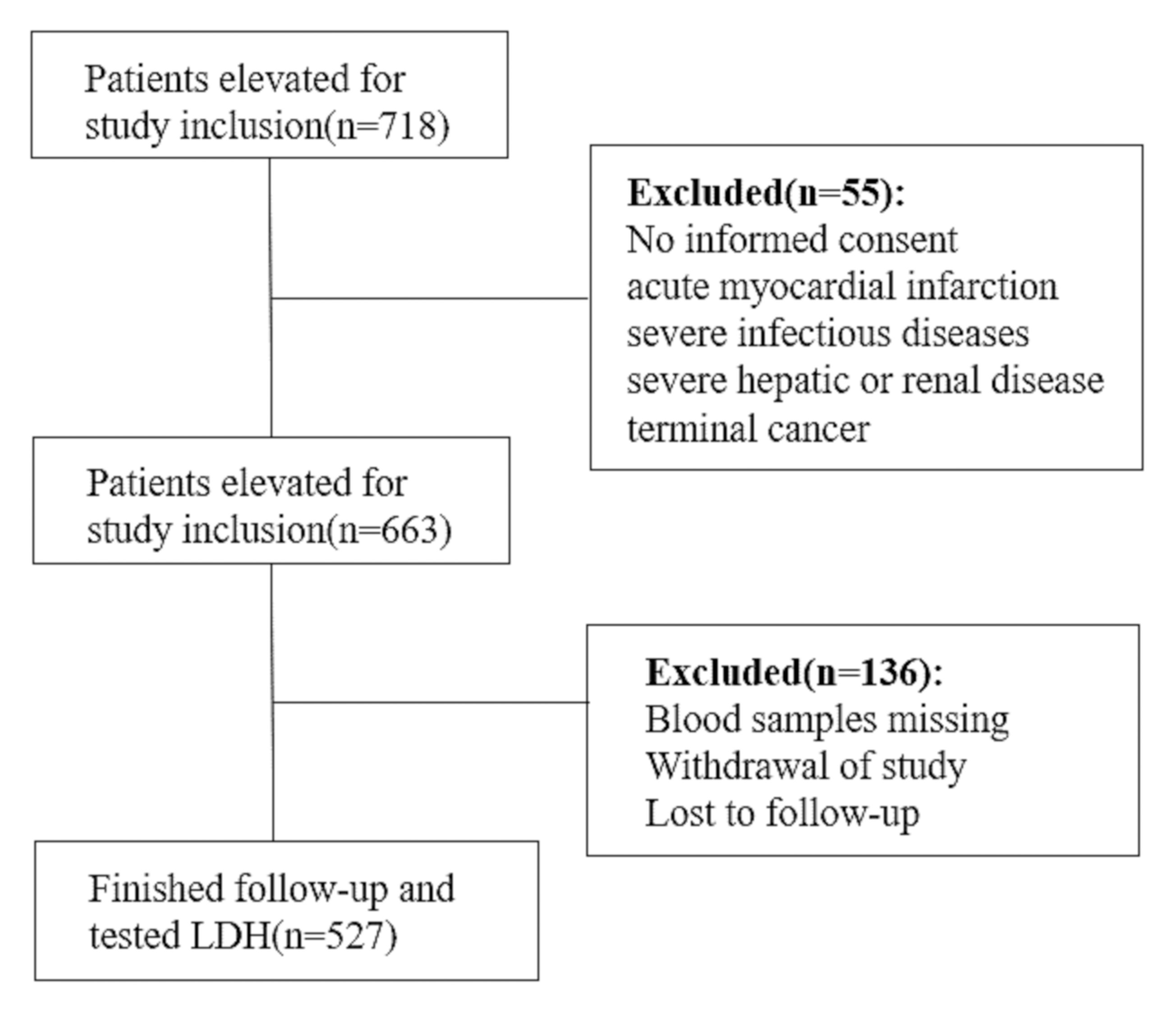

Supplement: Supplementary file 2 [file Image_1.TIF]
